# The Burden and Characteristics of Enteric Fever at a Healthcare Facility in a Densely Populated Area of Kathmandu

**DOI:** 10.1371/journal.pone.0013988

**Published:** 2010-11-15

**Authors:** Abhilasha Karkey, Amit Arjyal, Katherine L. Anders, Maciej F. Boni, Sabina Dongol, Samir Koirala, Phan Vu Tra My, Tran Vu Thieu Nga, Archie C. A. Clements, Kathryn E. Holt, Pham Thanh Duy, Jeremy N. Day, James I. Campbell, Gordon Dougan, Christiane Dolecek, Jeremy Farrar, Buddha Basnyat, Stephen Baker

**Affiliations:** 1 Oxford University Clinical Research Unit, Patan Academy of Health Sciences, Lagankhel, Kathmandu, Nepal; 2 Oxford University Clinical Research Unit, Wellcome Trust Major Overseas Programme, The Hospital for Tropical Diseases, Ho Chi Minh City, Vietnam; 3 The MRC Centre for Genomics and Global Health, Oxford, United Kingdom; 4 School of Population Health, University of Queensland, Brisbane, Australia; 5 Department of Microbiology and Immunology, The University of Melbourne, Melbourne, Australia; 6 Wellcome Trust Sanger Institute, Cambridge, United Kingdom; Menzies School of Health Research, Australia

## Abstract

Enteric fever, caused by *Salmonella enterica* serovars Typhi and Paratyphi A (*S*. Typhi and *S*. Paratyphi A) remains a major public health problem in many settings. The disease is limited to locations with poor sanitation which facilitates the transmission of the infecting organisms. Efficacious and inexpensive vaccines are available for *S*. Typhi, yet are not commonly deployed to control the disease. Lack of vaccination is due partly to uncertainty of the disease burden arising from a paucity of epidemiological information in key locations. We have collected and analyzed data from 3,898 cases of blood culture-confirmed enteric fever from Patan Hospital in Lalitpur Sub-Metropolitan City (LSMC), between June 2005 and May 2009. Demographic data was available for a subset of these patients (n = 527) that were resident in LSMC and who were enrolled in trials. We show a considerable burden of enteric fever caused by *S*. Typhi (2,672; 68.5%) and *S*. Paratyphi A (1,226; 31.5%) at this Hospital over a four year period, which correlate with seasonal fluctuations in rainfall. We found that local population density was not related to incidence and we identified a focus of infections in the east of LSMC. With data from patients resident in LSMC we found that the median age of those with *S*. Typhi (16 years) was significantly less than *S*. Paratyphi A (20 years) and that males aged 15 to 25 were disproportionately infected. Our findings provide a snapshot into the epidemiological patterns of enteric fever in Kathmandu. The uneven distribution of enteric fever patients within the population suggests local variation in risk factors, such as contaminated drinking water. These findings are important for initiating a vaccination scheme and improvements in sanitation. We suggest any such intervention should be implemented throughout the LSMC area.

## Introduction

Enteric fever is a term given to the human diseases caused by invasive *Salmonella* serovars, namely, *Salmonella enterica* serovar Typhi (*S.* Typhi) and the various *Salmonella enterica* serovar Paratyphi serovars (*S.* Paratyphi A, B and C) [1], [2]. *S*. Typhi generally predominates with respect to the aetiology of enteric fever, yet *S.* Paratyphi A is recognized as an escalating cause [3], [4], [5]. Enteric fever disproportionately affects children and is characterized by persistent fever, malaise and may cause a protracted illness lasting several weeks. The disease is seldom fatal, but some patients can develop life threatening complications, including hypotensive shock and perforation of the intestine [2], [6]. The organisms are transmitted via the faecal-oral route. Therefore, the global distribution of the disease is limited to areas with poor standards of hygiene and sanitation, which facilitate transmission [7]. Cases in industrialized countries are few and are invariably restricted to those returning from travel to endemic areas [8], [9]. Enteric fever remains a public health concern in endemic urban locations, exacerbated by the circulation of diverse genotypes and antimicrobial resistant organisms [10], [11], [12].

The current global incidence calculations for enteric fever are based on population-level data available from ten countries; these figures estimated that in 2000 there were over 21 million new cases and >200,000 deaths due to *S.* Typhi and >5 million new infections caused by *S*. Paratyphi A [13]. Very few countries with endemic and epidemic disease have implemented typhoid immunization programs despite the global incidence and current WHO recommendations to employ vaccines based upon knowledge of the local epidemiological situation [14], [15], [16]. Therefore, for an effective control program (vaccination or otherwise), in locations such as Kathmandu, it is essential to understand the local distribution and burden of enteric fever. Knowing the proportion of disease due to *S*. Typhi and *S*. Paratyphi A is also significant for intervention [17], as these serovars may have subtle differences in their modes of transmission and may necessitate differing control measures [18], [19]. *S*. Paratyphi A is a particular challenge as there is currently no licensed vaccine, and a vaccine study in Kolkata, India demonstrated that whilst the Vi vaccine was highly protective for *S*. Typhi infections, there was no cross-protection against *S*. Paratyphi A [20], [21]. There is a paucity of data regarding *S*. Typhi and *S*. Paratyphi A infections originating from Nepal and in particular Kathmandu, which is considered to have a high incidence of enteric fever [22].

Our previous work at Patan Hospital in Lalitpur Sub-Metropolitan City (LSMC), an urban district of Kathmandu, includes a retrospective and a prospective clinical assessment of enteric fever patients and a randomized controlled treatment trial [23], [24], [25]. The purpose of this work was to calculate the burden of enteric fever attributable to *S*. Typhi and *S*. Paratyphi A at Patan Hospital in Kathmandu and estimate incidence in the population residing in LSMC. To address this aim, data were obtained from two sources; retrospective data on culture-confirmed enteric fever patients between June 2005 and May 2009 were extracted from the diagnostic microbiology database at Patan Hospital and prospective demographic data were obtained from patients enrolled three clinical trials conducted over the same time period. Here we describe the epidemiological characteristics and the aetiology of enteric fever in patients presenting to Patan Hospital in LSMC, Kathmandu between 2005 and 2009.

## Results

### Enteric fever at Patan Hospital; June 2005 to May 2009

**Figure 1:**
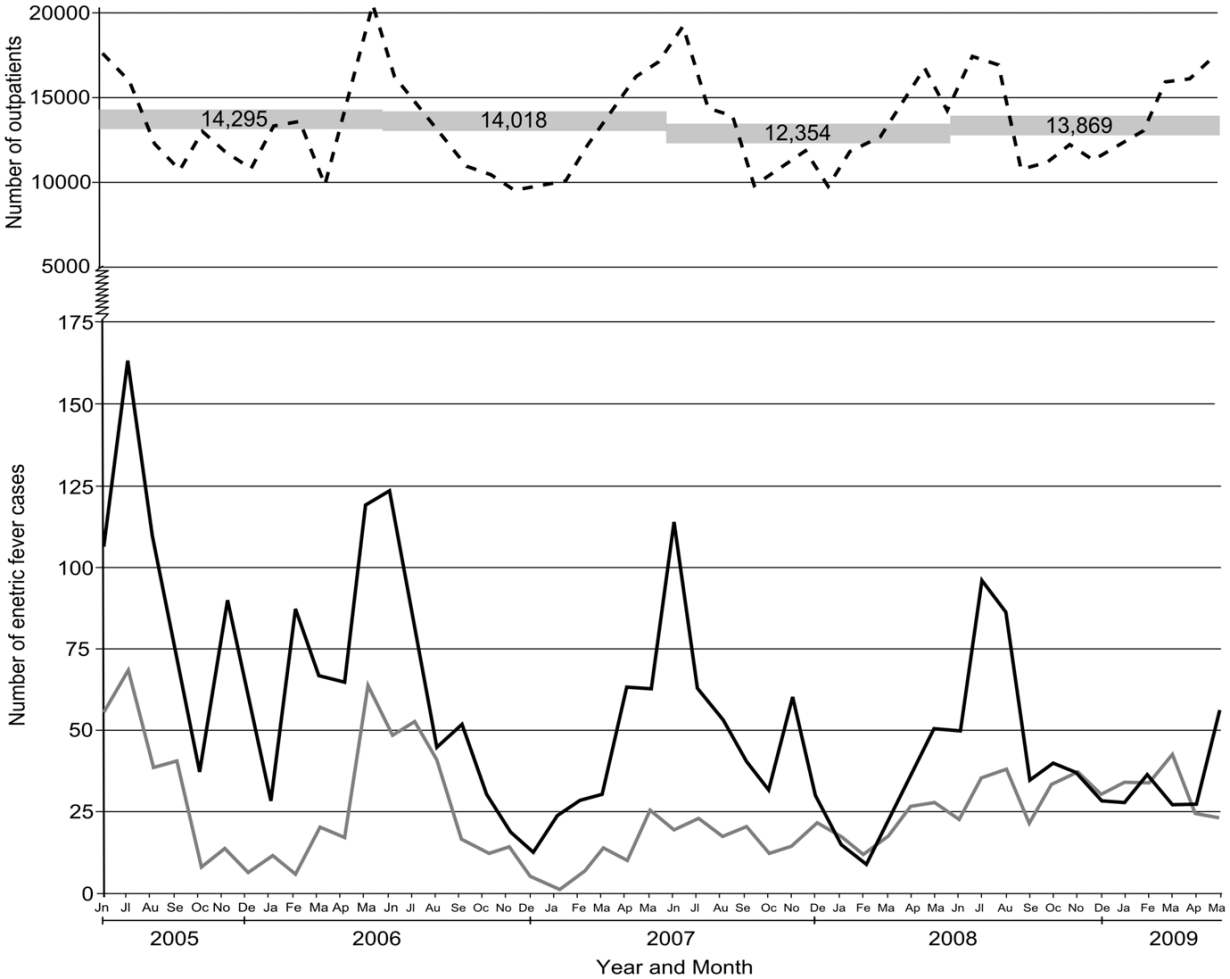
Enteric fever case burden in patients attending Patan Hospital, 2005–2009. Plot showing the number of culture positive enteric fever cases at Patan Hospital per month between June 2005 and May 2009 (solid black line; *S*. Typhi and solid grey line; *S*. Paratyphi A). The broken black line shows the number of patients attending the outpatient department per month over the same period, with numbers in grey boxes equating to the annual number of blood cultures performed.

The number of enteric fever patients presenting to Patan Hospital by month, the number of outpatient attendees and the number of blood cultures performed are shown in Figure 1. From June 2005 to May 2009, there were 54,536 blood cultures performed at Patan Hospital, of which 3,898 (7.15%) were positive for an invasive *Salmonella*. Of the 3,898 invasive *Salmonella* identified, 2,672 (68.5%) were *S*. Typhi and 1,226 (31.5%) were *S*. Paratyphi A, no *S*. Paratyphi B or C were observed. Over the four year period there was an overall decline in enteric fever cases (despite a slight increase from 2007 to 2008). This decline was not related to the number of blood cultures performed (14,295 in 2005 and 13,869 in 2008) or the number of patients attending the outpatients departments (relative risk = 0.82 per year, 95% CI 0.75 to 0.90, *p*<0.001)(Figure 1). Indicating that the observed decline in the number of enteric fever patients during this period is probably not an artefact of overall healthcare-seeking behaviour.

The overall decline in enteric fever appears to be dominated by an annual decrease in *S*. Typhi cases (728 in 2006, 605 in 2007 and 511 in 2008). The increase in enteric fever from 2007 to 2008 (813 in 2007 and 857 in 2008) was due to an increase in *S*. Paratyphi A cases (192 in 2007 and 324 in 208)(Figure 1). From October 2008 to April 2009 the number of *S*. Paratyphi A cases was almost equivalent to the number of *S*. Typhi cases. Annual peaks in enteric fever cases correlated with the peak attendance in the outpatients department, suggesting seasonal variation of febrile disease in this location.

### The seasonal distribution of enteric fever cases

**Figure 2:**
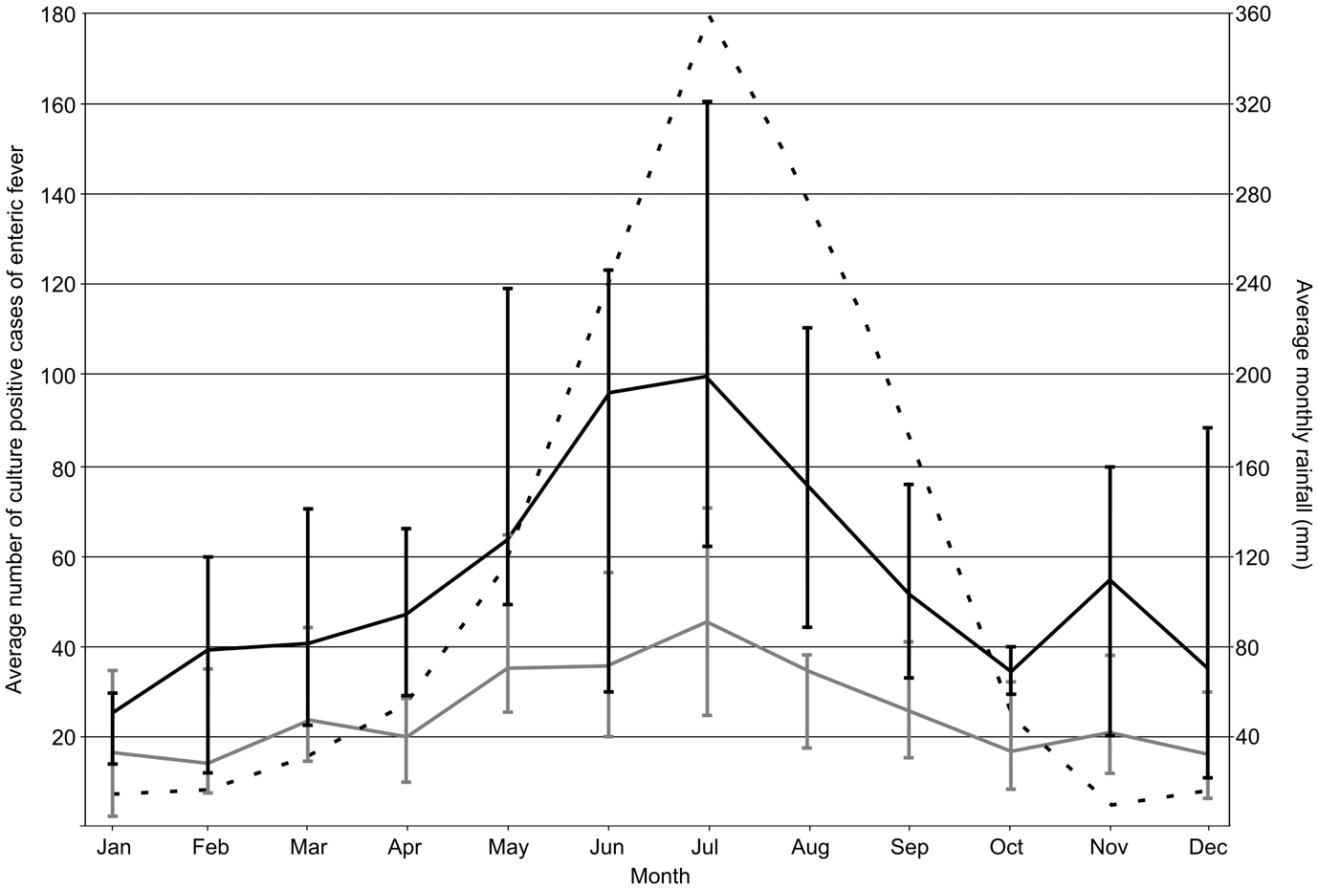
The seasonal distribution of enteric fever patients at Patan Hospital. Plot of the seasonal distribution of enteric fever patients at Patan Hospital in Kathmandu. The average number of *S.* Typhi cases (black line) and *S.* Paratyphi A cases (grey line) for each month of the year was calculated over the period June 2005–May 2009. Vertical lines represent the range over the four year period for each month. The average monthly rainfall (mm) is shown by the broken black line and corresponds with the secondary y axis.

Figure 2 shows the average number of blood culture confirmed cases of *S*. Typhi and *S*. Paratyphi A per month at Patan Hospital over the four year period of study (monthly data were combined and divided by the four years of the study). There was a seasonal signal in the monthly frequency of enteric fever cases, which correlated with the average volume of rainfall per month over the same time period. The peak of the wet season in Kathmandu occurs in July, which corresponds with a peak in both *S*. Typhi (average number of cases in July; 100) and *S*. Paratyphi A cases (average number of cases in July; 45) at Patan Hospital. The rainfall subsides after August and between October and April an average of less than 56 *S.* Typhi cases and 24 *S.* Paratyphi A cases are observed per month. The average seasonal distribution is comparable for *S*. Typhi and *S*. Paratyphi A, implying a parallel relationship between enteric fever of both aetiologies to seasonal variation in rainfall (*p* = 0.91; χ^2^-test, 11 df).

### The incidence of enteric fever within LSMC

During the period from June 2005 to May 2009, we enrolled 527 patients that had a positive blood culture for either *S*. Typhi or *S.* Paratyphi A in clinical studies at Patan hospital. These enrolees constituted 13.5% (527/3,898) of all the blood culture positive enteric fever patients at Patan hospital over the same period. The remainder of enteric fever patients were not enrolled in clinical studies; this was dependent on the criteria of enrolment (see methods) and additional factors, including, the time of admission to hospital, admission during periods when clinical studies were not running, and residence outside of LSMC.

**Figure 3:**
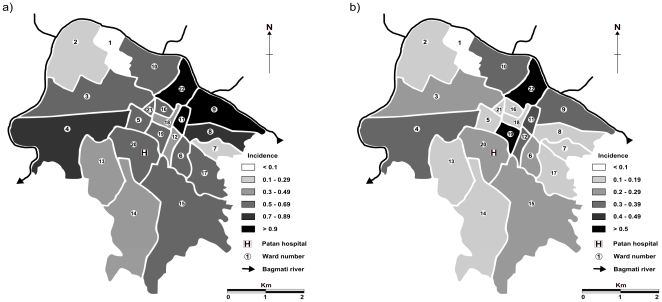
The distribution of enteric fever cases in LSMC. Maps depicting the average annual incidence of enteric fever per 1,000 population in the 22 wards (numbered) that constitute the Lalitpur Sub-Metropolitan City (LSMC), based on enteric fever cases enrolled in clinical trials at Patan Hospital between June 2005 and May 2009 for (a) *S*. Typhi and b) *S*. Paratyphi A). Patan Hospital is located in ward 20 and is highlighted. Ward population figures are presented in Table 1.

All 527 enrolled patients were resident in the 15.43 km^2^ that constitute the 22 wards of LSMC. These data were categorized by ward and using population figures for LSMC wards as a denominator, the average annual incidence of *S*. Typhi, *S*. Paratyphi A and total enteric fever in each of the 22 wards and overall in LSMC was calculated over this four year period and is reported in Table 1. The three wards with the highest incidence of enteric fever during the study period (wards 9, 22 and 11; incidence 1.66, 1.53 and 1.24 per 1,000 residents per year, respectively) are neighbouring and located in the east of the City, bordering the Bagmati River (Figure 3). Ward 22 had both the highest incidence of *S*. Typhi and *S*. Paratyphi A and ward 1 had the lowest (Figure 3). Despite enteric fever following a human-to-human transmission route, there was no correlation between population density and enteric fever incidence in the 22 wards (*p* = 0.857, r = 0.037, Spearman's *ρ*). There was also no association between ward-level incidence in this group of patients and the average distance of patients' residence to Patan Hospital (*p* = 0.1706, r = −0.29757, Spearman's *ρ*). *S*. Typhi was predominant over *S*. Paratyphi A in all but two wards and the ratio of *S*. Typhi to *S*. Paratyphi A ranged from 1∶1 to 5.67∶1. We calculated the average incidence of enteric fever in LSMC to be 3.78 cases per 1,000 households per year over the period 2005–2009. Similar to the population incidence of enteric fever, wards 9, 22 and 11 had the highest household incidences.

**Table 1:** The average annual incidence of enteric fever cases in LSMC, by ward of residence.

Ward	Population^a^	Population/ha^a^	*S*. Typhi		*S.* Paratyphi A		Total		Ratio^c^	Distance (km) d	Incidence/1,000
			Number	Incidence^b^	Number	Incidence^b^	Number	Incidence^b^			Households/year
**1**	7,096	171.32	2	0.07	2	0.07	4	0.14	1.00	2.17	0.60
**2**	10,459	80.61	11	0.26	4	0.10	15	0.36	2.75	2.20	1.65
**3**	10,637	71.86	23	0.54	9	0.21	31	0.75	2.56	1.55	3.38
**4**	10,971	60.72	37	0.84	14	0.32	51	1.16	2.64	1.66	5.05
**5**	6,573	93.18	15	0.57	5	0.19	20	0.76	3.00	0.30	3.58
**6**	6,352	249.49	14	0.55	6	0.24	20	0.79	2.33	0.63	3.83
**7**	6,408	268.79	6	0.24	3	0.12	9	0.35	2.00	1.07	1.73
**8**	7,355	165.80	21	0.72	4	0.14	25	0.85	5.25	1.04	4.45
**9**	8,135	105.35	44	1.35	10	0.31	54	1.66	4.40	1.15	7.93
**10**	5,430	66.95	12	0.55	8	0.37	20	0.92	1.50	1.75	4.10
**11**	4,238	338.50	17	1.00	4	0.24	21	1.24	4.25	0.92	6.73
**12**	5,677	430.40	10	0.44	8	0.35	18	0.79	1.25	0.43	3.98
**13**	6,553	68.79	11	0.42	5	0.19	16	0.61	2.20	1.14	2.85
**14**	11,530	62.45	15	0.33	7	0.15	22	0.48	2.14	1.44	2.20
**15**	11,352	46.66	27	0.60	9	0.20	36	0.79	3.00	1.26	3.35
**16**	5,294	540.20	12	0.57	4	0.19	16	0.76	3.00	1.00	4.05
**17**	6,693	118.08	17	0.64	3	0.11	20	0.75	5.67	0.94	3.33
**18**	6,915	539.39	11	0.40	3	0.11	14	0.51	3.67	0.66	2.73
**19**	6,048	345.21	12	0.50	12	0.50	24	0.99	1.00	0.42	4.75
**20**	6,519	328.58	17	0.65	7	0.27	24	0.92	2.43	0.66	4.15
**21**	4,249	452.99	11	0.65	3	0.18	14	0.82	3.67	0.83	3.88
**22**	8,513	184.66	34	1.00	18	0.53	52	1.53	1.89	1.16	6.88
**Total/Average**	**162,997**	**217.73**	**379**	**0.59**	**148**	**0.23**	**527**	**0.82**	**2.80**	**1.11**	**3.78**

a Data from the 2001 Nepal census [36].

b Incidence calculated as cases per 1,000 population per year.

c Ratio of *S*. Typhi to *S*. Paratyphi A per ward.

d Average distance from Patan Hospital to patients' location of residence.

### Age and gender distribution of enteric fever patients

**Figure 4:**
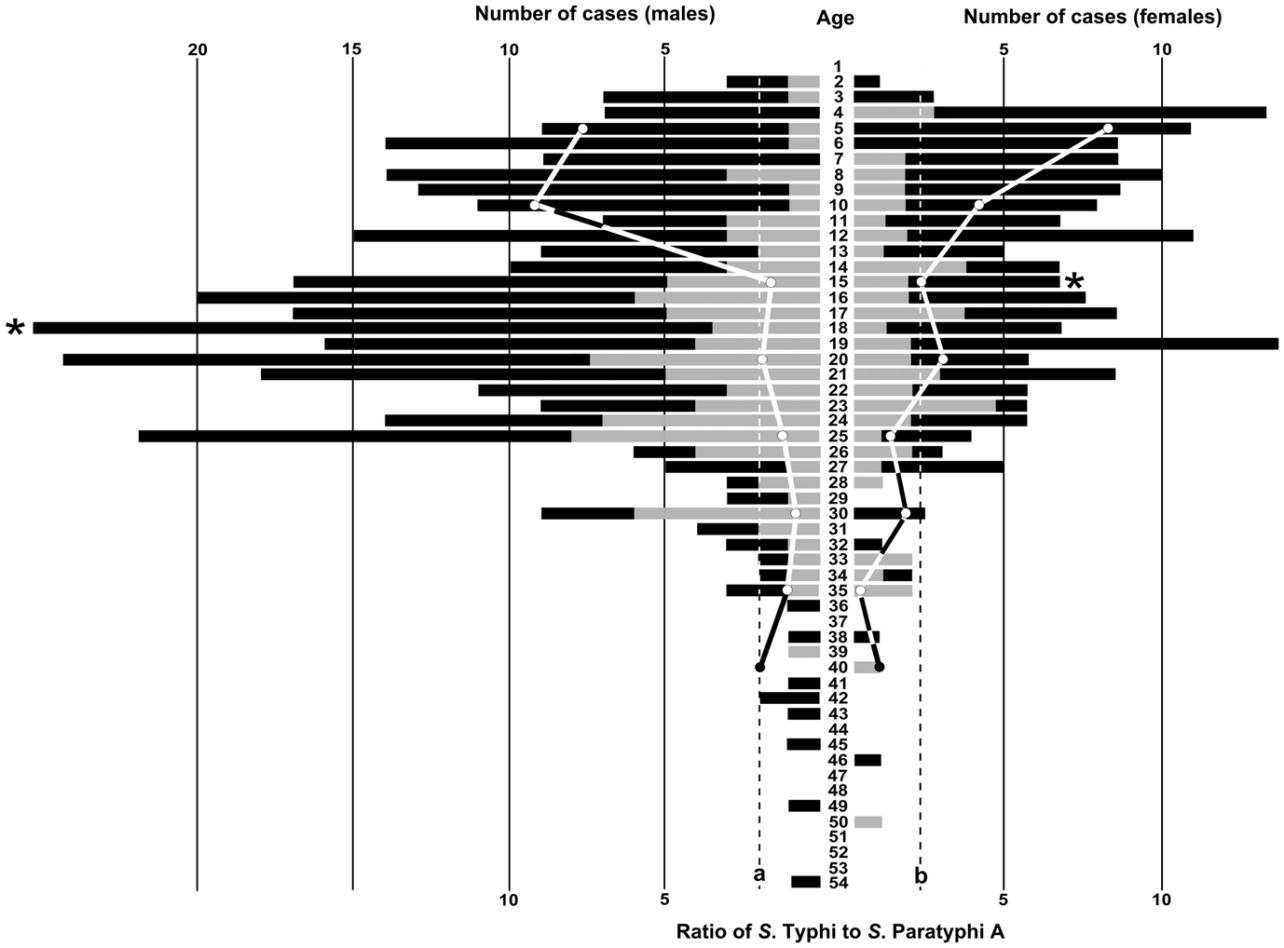
The age distribution of a subset of enteric fever cases at Patan Hospital, LSMC. A double sided bar chart showing the age distribution (central scale; y axis) of blood culture positive enteric fever cases enrolled in clinical trials at Patan Hospital in LSMC between June 2005 and May 2009 (n = 527). Bar sizes correspond to the number of patients (upper x axis) of each sex (left; male and right; female). Cases that were culture positive for *S*. Typhi are shown in dark shading, those that were culture positive for *S*. Paratyphi A are shown in light shading. The ratio of culture positive *S*. Typhi to *S*. Paratyphi A cases over the period of investigation in each five year age group for males and females are shown by the solid lines with solid circles (lower x axis). The overall ratio of *S.* Typhi to *S*. Paratyphi A was 2.69∶1 in males (shown by the black broken line a) and 2.73∶1 in females (shown by the black broken line b). The median age of the male and female patients is shown by the asterisk.

The distribution of all the 527 enrolled enteric fever cases by age and gender is shown in Figure 4. Sixty-four percent of all enteric fever cases were male (male∶female, 1.76∶1). The number of male cases of enteric fever exceeded female cases across almost all age groups and, in particular, among young adults aged between 15 and 30 years. The median age of male cases (18 years) was higher than that of female cases (15 years), *p* = 0.003. The median age of *S.* Typhi cases (16 years) was significantly lower than that of *S.* Paratyphi A cases (20 years), *p*<0.0001. In both males and females, *S*. Typhi was the predominant etiological agent across all age groups, which is consistent with our observation that *S*. Typhi is more prevalent than *S*. Paratyphi A in this location. Notably, among children under the age of 10 years, the ratio of *S*. Typhi to *S*. Paratyphi A exceeded 8∶1, then declined with age (Figure 4).

## Discussion

Enteric fever is a persistent problem in Kathmandu and in other urban locations in South and South-East Asia [7], [26]. Here we present some epidemiological features of enteric fever in LSMC. We found a considerable burden of *S*. Typhi and *S*. Paratyphi A infections at Patan Hospital, which fluctuates with seasonal variation in rainfall. From available data on patients enrolled in clinical trials, we have calculated a conservative average annual enteric fever incidence in LSMC over a four-year period and investigated the age distribution and spatial variation in disease incidence. The significance of these findings is that this area of Kathmandu would be suitable for programmatic use of typhoid vaccines for controlling the disease as suggested by the WHO [15], as demonstrated in studies conducted in South Asia [27], [28].

Our findings, however, have limitations as they are based upon available data from those people with enteric fever receiving a blood culture that present to a single health care facility in LSMC. Additionally, the age distribution, incidence and locality data are taken from a subset of patients entering clinical trials. Therefore, we present a snapshot of some of the epidemiological features of enteric fever in this location, and we recognize that the subset of patients on which our analysis is based may not be fully representative of the enteric fever patient population of LSMC as a whole. However, in the absence of prospective population-based surveillance data, these findings from a large dataset provide insight into the patterns of enteric fever within an urban population and will lead to more detailed epidemiological studies within this population.

We observed that the proportion of *S*. Typhi to *S*. Paratyphi A cases fluctuated over the four year study period. There was a sequential annual decrease in *S.* Typhi and an increase in *S.* Paratyphi A from June 2005 to May 2009, with an overall decrease in total enteric fever cases over the four year period. Previous studies have reported that an increasing fraction of the enteric fever cases in this population were caused by *S*. Paratyphi A between 1999 and 2003 [23], it appears that this trend is continuing. We also observed variation in the proportion of *S*. Typhi to *S*. Paratyphi A at the ward level and by patient age. These observations may be explained by differing transmission factors between these two pathogens, as well as a difference in the overall burden of the two serovars. *S*. Paratyphi A incidence is lower than *S*. Typhi, suggesting that exposure to *S*. Paratyphi A is lower than to *S*. Typhi. We would, therefore, expect the average age of patients infected with *S*. Paratyphi A infections to be higher, since lower exposure implies a longer time to acquired immunity; this is consistent with what we observed. Other authors have reported independent risk factors for transmission of *S*. Typhi and *S*. Paratyphi A within the same area[19], and our data showing differences in the ward level distribution of the two serotypes are consistent with this. The observed decrease in *S*. Typhi at Patan Hospital over the four year period may be due to changing health care seeking behaviour or increasing immunity within a highly exposed population [29].

Enteric fever is considered to be a particular public health issue in children [30]. Although we found a substantial number of enteric fever cases in children, we observed a disproportionate number of enteric fever cases in young male adults among the subset of patients enrolled in clinical trials. Here, one must consider potential gender and age bias in study recruitment, as not all patients with enteric fever may have been equally likely to enrol in the clinical trials. While we cannot draw robust conclusions regarding the age and gender distribution of enteric fever patients in LSMC, some observations warrant consideration. The majority of cases were males aged between 16 and 30 years. Kathmandu has a large predominately male transient workforce, i.e. those coming from other areas of Nepal for employment. A demographic study of the Nepalese population in 2007 found that 37% of households had at least one person that had travelled away from the household for employment within Nepal at some time in the previous 12 months [31]. Men were found to be nearly three times as likely as women to have migrated for employment and movement was mainly limited to those between the age of 15 and 30 years [31]. This observation is intriguing and if these data are representative the disproportionate number of infected males may be attributable to increased risk of exposure (related to behaviour and/or living conditions) or lack of immunity due to a lack of previous exposure.

We found some variation in the spatial distribution of the enrolled enteric fever patients, with a major focus of infections in the east of the City. However, there was no direct relationship between the population density or distance from the hospital and the incidence of enteric fever cases in individual wards, suggesting the local environment plays an important role in transmission. We calculated a minimum estimate of average annual incidence of enteric fever in LSMC of 0.82 per 1,000 residents per year. This frequency makes LSMC an area of medium incidence according to the guidelines outlined by Crump *et al.* [13] and equivalent to the (*S*. Typhi) incidence calculated from population surveillance in Jakarta, Indonesia [32]. Our calculation is based on those with culture confirmed enteric fever enrolled in clinical studies attending one health care facility. Therefore, our figures are likely to be highly conservative and we estimate (based on the proportion of total outpatient attendees living in LSMC) it may be at least five times greater than this figure. This makes LSMC an area of high enteric fever incidence and enteric fever is likely to place a considerable burden on the health care system in the study area.

We propose that environmental factors play a significant role in the transmission of the infecting organisms; this is supported by the association of incidence with rainfall, an association that has been observed to a lesser extent in Vietnam [30]. Preliminary data from an ongoing study show a correlation between increased rainfall and increased faecal contamination of the water supply, which has been identified in enteric fever regions of Pakistan and Vietnam [33], [34]. Preventive measures for enteric fever must include improvements of water supplies and sanitation facilities. An improvement in infrastructure requires substantial investment and seems an unachievable short term objective in Kathmandu. Nepal remains among the poorest and least developed countries in Asia with almost one third of its population living below the poverty line as defined by the world bank [35]. Health education, case identification and appropriate treatment and vaccination may be the only solution to ensure the incidence of this disease continues to decline. Our study supports previous findings that enteric fever caused by *S*. Typhi is largely a childhood disease; therefore, school age children are an obvious target group for a vaccination program. However, further investigation is also required of the high case burden we observed in young adult males, to determine whether particular risk group such as transient workers should be targeted in prevention and control activities. Young adult males were relatively more likely to be infected by *S*. Paratyphi A for which there is no vaccine, an *S*. Paratyphi A vaccine would be valuable tool to prevent infection and control transmission.

The median age of enteric fever may be higher in LSMC than in other locations and we suggest that tackling the disease in school-age children and the transient male population may be key to controlling the ongoing transmission of enteric fever in this densely-populated area. The uneven spatial distribution of enteric fever incidence within the Patan Hospital catchment population suggests local variation in risk factors, and control measures should be implemented throughout the LSMC area. In the absence of prospective population-based surveillance, these findings from a large clinical dataset provide significant information on the distribution of enteric fever within an urban population.

## Materials and Methods

### Ethics statement

This study was conducted according to the principles expressed in the Declaration of Helsinki. This work, including the mapping of the patients' residence and the use of hospital data, was approved by the relevant institutional ethical review boards including Patan Hospital, The Nepal Health Research Council and Oxford Tropical Research Ethics Committee (OXTREC). Enrolees to all three clinical trials were required to provide written informed consent for the collection of samples and subsequent analysis. In the case of children this was performed by the parent or guardian.

### Study site

The Kathmandu valley is in the central area of Nepal and is home to 20% of Nepal's urban population [36]. The majority of the population lives in the City of Kathmandu, which has a population of approximately 1.5 million [36]. Lalitpur Sub-Metropolitan City (LSMC; also known as Patan) is situated on an elevated plateau of land, separated from the metropolitan City of Kathmandu to the north by the Bagmati River and is one of the three urban districts that constitute the Kathmandu valley. LSMC is comprised of 22 municipal wards with a land area 15.43 km^2^. According to the 2001 Nepalese census, LSMC had a population of 162,991 living in 68,922 households. Fifty-one percent of the population is male and the area is densely populated with an average density of 10,560 people per km^2^ (105.6 per hectare) [36]. The population of LSMC is generally poor, overcrowding in residential buildings is common and people obtain their drinking water from numerous sunken wells and municipal supplies.

Patan Hospital is located in LSMC and is the only hospital in the sub-metropolitan City. It is a 318 bed government hospital providing emergency and elective outpatient and inpatient services. There are some 200,000 outpatient visits, 35,000 emergency visits and 15,000 admissions annually. Patan Hospital is the only hospital in LSMC, yet there are numerous private physicians clinics where patients may seek advice and clinical diagnosis for febrile diseases, such as enteric fever and antimicrobials are available in the community. Therefore, patients attending Patan Hospital may only represent the most severe end of the spectrum of enteric fever as many patients may seek local advice or self-treat. There has been no government implementation of a typhoid vaccine program in this area. A generic typhoid Vi vaccine is available for purchase in some health care settings (including Patan Hospital), yet there is limited uptake in the community.

### Study population

At Patan Hospital, all febrile patients suspected of having bacteraemia have a blood culture performed. Retrospective data extracted from the microbiology database included the total number of blood cultures performed monthly and the number that were culture positive for *S.* Typhi or *S*. Paratyphi A. These data were used to plot the case burden and seasonal trends at this single health care facility. Data were also extracted from the hospital database on the total number of people attending the outpatients department over the same period.

The demographic data analyzed for this study were collected from patients enrolled in three consecutive randomized controlled trials at Patan Hospital for the treatment of uncomplicated enteric fever. These trials were gatifloxacin versus cefixime (ISRCTN75784880) [25], gatifloxacin versus chloramphenicol (ISRCTN53258327) and gatifloxacin versus ofloxacin (ISRCTN53258327). The population enrolled in clinical trials were patients who presented to the outpatient or emergency department of Patan Hospital (as above) between June 2005 and May 2009 and met enrolment criteria. The clinical trials enrolees were, therefore, a proportion of the total enteric fever cases at this health care facility. Patients were eligible to enter the studies if they had clinically diagnosed enteric fever (including a temperature of >38°C). Other inclusion criteria were that patients must be aged between 2 and 65 years, able to stay in the city for the duration of the treatment, not known to have contraindications to either cephalosporins or fluoroquinolones, and willing to give informed written consent to take part in the study. Patients who had received a third generation cephalosporin, fluoroquinolone or macrolide in the week prior to presentation to Patan Hospital were also excluded. The enrolment criteria were consistent for all three trials and are described in greater detail in Pandit *et al*. [25].

### Microbiological culture

Samples of 10 ml of anti-coagulant blood were collected in EDTA tubes from febrile patients over the age of 12 years and 5 ml from those 12 years of age or younger. For the culture of *Salmonella* serovars, 6 ml and 3 ml of blood were used for those over 12 years and those 12 years of age or younger, respectively. The EDTA blood was inoculated into 30–50 ml of media containing tryptone soya broth and sodium polyethanol sulphonate. The media with the EDTA blood was incubated at 37°C and examined daily for bacterial growth over a seven day period. If growth was observed, the media was sub-cultured onto MacConkey agar media to isolate invasive *Salmonella* serotypes. Any colonies presumptive of *S*. Typhi or *S*. Paratyphi were identified using standard biochemical tests and serotype-specific antisera (Murex Biotech, Dartford, England).

### Data analysis

A case of enteric fever was defined as any patient with a positive blood culture for *S*. Typhi or *S*. Paratyphi A attending Patan hospital. A primary analysis of temporal and seasonal trends in the enteric fever case burden was performed on data from patients with enteric fever at Patan hospital. Secondary demographic analysis was performed on a subset of patients that were enrolled in clinical trials, who represented a proportion of the total enteric fever cases attending the Hospital. All patients enrolled in clinical trials were resident in LSMC. The data from those enrolled in clinical trials was combined with population data, obtained from the 2001 Nepalese census [36], to calculate a minimum estimate of the incidence of enteric fever in LSMC per 1,000 persons per year and per 1,000 households per year. Data on the average monthly rainfall between 2005–2009 were obtained from the meteorological station at Kathmandu airport.

We tested whether there was a linear trend over time in the monthly rate of enteric fever cases using a Poisson regression with the (log-transformed) monthly number of outpatient admission as an offset. These data were adjusted for over dispersion using quasi-likelihood.The Chi squared test was used to compare the seasonal distribution of *S*. Typhi and *S*. Paratyphi A cases. Spearman's rank correlation coefficient was used to investigate the statistical dependence between population density or the average distance of patients' residence from Patan Hospital and the ward-level incidence of enteric fever. A 2 tailed t-test was used to compare the age distribution of male and females with enteric fever. All statistical analyses were performed in R (www.r-project.org).

## Supporting Information

Alternative Language Abstract S1 Translation of the Abstract into Nepalese by Abhilasha Karkey.
(0.03 MB DOC)

Checklist S1 STROBE checklist.
(0.05 MB PDF)
