# Supplementary material for: The Burden and Characteristics of Enteric Fever at a Healthcare Facility in a Densely Populated Area of Kathmandu
Source: PLoS One. 2010 Nov 15;5(11):e13988. doi: 10.1371/journal.pone.0013988 (PMC2981554; doi:10.1371/journal.pone.0013988)
Supplement: Alternative Language Abstract S1 — Translation of the Abstract into Nepalese by Abhilasha Karkey. (0.03 MB DOC) [file pone.0013988.s001.doc]

सारंश

पृष्ठ भुमि

टाइफाइड ज्वरो सालमोनेला इन्टेरिका सेरोभार्स टाइफी (Salmonella enterica serovars Typhi) र प्याराटाइफी ए (Paratyphi A) ले गर्दा हुन्छ, तर यो रोग लगातार रुपमा बेवास्ता हुने गरेको पाइएको छ। मुलत यो रोग कम-सफाई व्यवस्था भएको ठाऊँमा बढी फैलने गर्दछ। सस्तो र प्रभावकारी खोप हुदाहुदैपनि यसको प्रयोग भने रोगको रोकथाममा हुन सकेको छैन। रोगबाट हुन सक्ने हानी र यसको जनस्वास्थमा हुने असरका बारेमा जानकारीको कमीहुनुले पनि खोप लगाउने प्रवृत्तिमा कमी आएको हामीले काठमांण्डौमा पाएका छौ।

**प्रकृया**

हामीले पाटन अस्पताल, ललितपुर उप-महानगरपालिकामा सन २००५ जुन महिना देखि सन् २००९ मे महिना सम्ममा आएका जम्मा ३,३९८ बिरामीको आंकडा लिएर त्यसको विश्लेषण गरेका छौ। ललितपुर उप-महानगरपालिकामा बसोबास गर्ने र रोगबिषयक अनुसन्धानमा समेटिएका (५२७ जना) बिरामीलाई जनसंखिय आंकडाका रुपमा लिएको छ।

*प्रमुख खोज*

सालमोनेला टाइफी (२,६७२: ६८.५ %) र प्याराटाएफी ए (१,२२६; ३१.५%) को कारणबाट हुने *टाईफाईड ज्वरोको उल्लेखनिय भार यसै अस्पतालको बिगतका ४ बर्षको आंकडालाइ लिएर हामीले देखाएका छौ र यसलाई मौसमी परिवर्तन ( बर्षा) संगँ दाजेर पनि हेरेका छौ। ५२७ जना विरामीको आंकडा अनुसार स्थानिय जनसंख्या वा धनत्व दुबैको, रोगीको संख्याको घटबढसगँ कुनै संबन्ध नरहेको र ललितपुरको पुर्वी भेगमा बढी संका्मण भएको पाइएको छ। उक्त बिरामीको आंकडा अनुसार एस.टाईफी हुने बिरामीको औसत उमेर १३ बर्ष छ जुन एस . प्याराटाईफि हुनेको औसत उमेर २० बर्ष भन्दा कम छ, साथसाथै जवान पुरुषहरुमा असमान रुपमा संक्रमण भएको पाइएको छ।*

*मह्त्व*

यो जनसंख्यामा आधारित अध्ययन नभएतापनि यस अध्ययनबाट प्राप्त नतिजाले यस एरियाको महामारी संबन्धी पद्दतिलाइ परिद्रष्य गरेको छ। स्थानिय जनसंख्यामा टाईफाईड बिरामीको असमान विभाजन र स्थानिय भिन्नतामा रोगको जोखिम देखाउछ।   यस अध्ययनबाट पत्ता लागेको तथ्यहरु टाईफाईड रोगको रोकथामको सान्दर्भिक उपायहरुको लागि महत्वपुर्ण छन् र हाम्रो सुझाव, यो ललितपुर उप-महानगरपालिकामा अबलम्बन हुनु पर्दछ।

लेखक सारंश

टाईफाईड ज्वरो मानिसमा हुने एक जटिल संक्रमण हो जुन सालमोनेला (सालमोनेला टाइफी र सालमोनेला प्याराटाइफी ए) ब्याटेरियाको कारणबाट हुने गर्दछ। यो जिवाणु पाचननलिमा आक्रमण गर्दछ र पछि गएर रक्त संचार प्रणालीमा छिर्दछ। यो रोग विशेशत आर्थिक रुपमा कमजोर र कम-सफाई व्वस्था भएको विकासोन्मुख समाजमा पाइन्छ, जहाँ सालमोनेलाको लगातार रुपमा प्रवाह हुने वातावरण श्रृजना भएको पाइन्छ। हाम्रो काठमाण्डौमा भएको अगिल्लो अध्ययनले दुबै सालमोनेला टाएफी र सालमोनेला प्याराटाएफी ए संक्रमण समान रहेको देखाउँछ। पाटन अस्पतालमा सन् २००५ देखि सन् २००९ सम्म भर्ना भएका बृहत संख्यामा टाईफाईड बिरामीको विश्लेषण गरिएको थियो। वातावरणीय परिवर्तनसँग रोगको आपतन निर्भर गर्दछ जुन बर्षायाममा बढी छ। यसका साथसाथै संक्रमणको स्थानलाई जनसंख्या, जनघनत्व र बढी विरामी रहेको स्थानले निर्धारण गर्दैन। परम्परागत रुपमा यो एक वाल्यकालमा हुने रोग जुन दुबै लिंगमा बराबर रुपमा हुने भन्ने सोचाइ थियो, तथापि हाम्रो आंकडाले १५-२५ बर्षका पुरुषहरुमा बढी भएको पाइएको छ। हाम्रो तथ्यहरुले किटाणुको संक्रमण र जनसंख्यामा यसको विभाजनबारे जानकारी प्रदान गरेको छ र भविष्यमा ध्यानदिनुपर्ने रोकथामका सरल उपायहरु पनि प्रष्ट greपार्न खोजेको छ
